# Reliability study for rib index

**DOI:** 10.1186/1748-7161-10-S1-O47

**Published:** 2015-01-19

**Authors:** Konstantinos C Soultanis, Konstantinos Tsiavos, Theodoros B Grivas, Nikolaos A Stavropoulos, Vasileios I Sakallariou, Andreas F Mavrogenis, Panayiotis J Papagelopoulos

**Affiliations:** 1 1st Orthopaedic Department, Athens University, Medical school, Greece; 2 Department of Orthopaedics and Traumatology, TZANEIO General Hospital of Piraeus, Piraeus, Greece

## Objectives

The Rib Index, (RI), extracted from the double rib couture sign (DRCS) on lateral spinal radiographs to evaluate rib hump deformity in IS patients has been earlier introduced (Grivas at al 2000). Although various papers using the RI have been published, no study of its reproducibility was reported. To estimate the variations of the RI in a number of a pair set of lateral chest radiographs of healthy volunteers. The hypothesis was that the RI should have minimal variability for each patient having successive lateral radiographs.

## Methods

70 randomized patients were initially included in the study. Each of these patients has two consequent chest lateral radiographs (LRs) at the radiological department of our hospital, by the same technician, during the course of treatment, (named A and B group of LRs). The radiation source - patient distance was constant. All LRs obtained in an incorrect position of the patient and LRs of those who underwent a thoracic intervention were excluded. In 49 patients A and B LRs were found to be suitable for assessment. The RI was calculated in both LRs of each patient. The statistical analysis included the paired t-test in the set of LRs, its correlation coefficient (R2), the intra- and inter-observer error using the formula (SD/V2)/2, where SD is this of the differences of the two sets of measurement (As-Bs). The SPSS v16 statistical package was used.

## Results

In the 49 pairs of LRs there was no statistical difference of the RI, (paired t-test p<0.314). The RI in the A and B group of LRs was perfectly correlated (correlation coefficient R = 0,924, p<0.0001). The intra-observer error was 0.0080 while the inter-observer error was 0.0213.

## Conclusion

The RI proves to be a reliable method to evaluate the thoracic deformity or the effect of surgical or conservative treatment on the IS rib-cage deformity (hump). IR is a simple method, a safe reproducible way to assess the rib hump deformity based on lateral radiographs, without the need for any other special radiographs and exposure to additional radiation.

